# Functional timing or rhythmical timing, or both? A corpus study of English and Mandarin duration

**DOI:** 10.3389/fpsyg.2022.869049

**Published:** 2023-01-20

**Authors:** Chengxia Wang, Yi Xu, Jinsong Zhang

**Affiliations:** ^1^Department of Speech, Hearing, and Phonetic Sciences, University College London, London, United Kingdom; ^2^School of Information Science, Beijing Language and Culture University, Beijing, China

**Keywords:** rhythm class hypothesis, compressibility, segment duration, syllable duration, timing

## Abstract

It has been long held that languages of the world are divided into rhythm classes so that they are either stress-timed, syllable-timed or mora-timed. It is also known for a long time that duration serves various informational functions in speech. But it is unclear whether these two kinds of uses of duration are complementary to each other, or they are actually one and the same. There has been much empirical research that raises questions about the rhythm class hypothesis due to lack of evidence of the suggested isochrony in any language. Yet the alleged cross-language rhythm classification is still widely taken for granted and continues to be researched. Here we conducted a corpus study of English, an archetype of a stress-timed language, and Mandarin, an alleged syllable-timed language, to look for evidence of at least a tendency toward isochrony when much of the informational use of duration is controlled for. We examined the relationship between segment and syllable duration and the relationship of syllable and phrase duration in the two languages. The results show that in English syllables are largely incompressible to allow stress-timing because segment duration is inflexible to allow variable syllable duration beyond its functional use. Surprisingly, Mandarin does show a small tendency toward both equal syllable duration and equal phrase duration. Additionally, the duration of pre-boundary syllables in English increases linearly with break index, whereas in Mandarin, the duration increase stops after break index 2, which is accompanied by the insertion of silent pauses. We conclude, therefore, timing and duration in speech are predominantly used for encoding information rather being controlled by a rhythmic principle, and the residual equal-duration tendency in the two languages examined here show exactly the opposite patterns from the predictions of the rhythm class hypothesis.

## 1. Introduction

Ever since the classic works of Pike (1945) and Abercrombie (1964a,b, 1967), it has been widely known that languages of the world are either stress timed, syllable timed or mora timed (Ramus et al., 1999). In a stress-timed language, inter-stress intervals are constant, hence, isochronous, whereas in a syllable-timed or mora-timed language, successive syllables or morae are equal in duration (Pike, 1945; Abercrombie, 1964a, 1967). Languages like English, Russian, Arabic, in fact, most Germanic and Slavonic languages, are deemed stress-timed, while French, Telugu, Yoruba, and most Romance languages are believed to be syllable timed (Pike, 1945; Ladefoged, 1975; Rubach and Booij, 1985), and languages like Japanese and Tamil are regarded as mora-timed (Port et al., 1987; Steever, 1987; Bertinetto, 1989).

Experimental investigations, however, have been unable to find evidence of “isochrony” in either stressed-timed, syllable-timed or mora-timed languages. For stress timing, time spans between primary stresses in English did not cluster around some average value (Shen and Peterson, 1962). In “The North Wind and the Sun” read by David Abercrombie, inter-stress intervals showed no marked regularity (Uldall, 1971). In the six languages examined by Roach (1982), stress-timed ones exhibited a wide range of percentage deviations in inter-stress intervals. In fact, a proportional relationship is found between the number of syllables and duration of inter-stress intervals. As the number of segments increases, foot duration showed a clear tendency to increase (O’Connor, 1968), and the relationship between the number of intervening unstressed syllables and the inter-stress interval for real words in sentence context is linear (Lea, 1974). For syllable timing, a twelve-syllable sequence in French is not twice as long as a six-syllable sequence (Wenk and Wioland, 1982). In Spanish, syllable duration varied with the complexity of syllable structure, stress and position (Borzone De Manrique and Signorini, 1983). Pointon (1980) argued that “Spanish has no regular rhythm in the sense of an isochronous sequence of similar events, be they syllables or stress.” For mora timing, Warner and Arai (2001) found no evidence of durational compensation in spontaneous speech in Japanese that would make morae equal in duration.

The rhythm class hypothesis, however, received renewed interest in the 1990s due to the proposal of various rhythm metrics, which use consonantal and vocalic variability to quantify the rhythm classes of languages. The main measurements are %V (the proportion of vocalic intervals in an utterance), ∆V (the standard deviation of vocalic intervals within an utterance), ∆C (the standard deviation of consonantal intervals within an utterance; Ramus et al., 1999), VarcoC (Standard deviation of consonantal intervals divided by mean and multiplies 100), VarcoV (Standard deviation of vocalic intervals divided by mean and multiplies 100; Dellwo and Wagner, 2003; Dellwo, 2006), and the pairwise variability indices nPVI and rPVI (Pairwise Variability Index in their measurements on successive vocalic and intervocalic intervals; Grabe and Low, 2002). Although a large number of studies have applied the rhythm metrics to different languages and even varieties of non-native accents (Dankovičová and Dellwo, 2007; Orourke, 2008; Mok, 2009; Nolan and Asu, 2009; Arvaniti, 2012), problems in the computation, their instability due to speech rate, speaking style, within-speaker variation and measurement uncertainty, and their failure to clearly separate languages into the alleged rhythm classes were criticized (Deterding, 2001; Gibbon, 2003; Bertinetto and Bertini, 2008; Knight, 2011; Arvaniti, 2012; Nolan and Jeon, 2014; Dellwo et al., 2015; White and Malisz, 2020). Most critically, all the rhythm metrics were proposed to differentiate languages based on various phonological properties. Thus even if some of them were able to separate languages as expected, they would have only validated the syllable structure and vowel reduction that are already well known, without addressing whether it is relevant in terms of syllable or stress timing.

It has also been suggested that rhythm is a perceptual phenomenon rather than a fact of speech production (Nakatani et al., 1981; Arvaniti, 2009; Kohler, 2009), and that “we hear speech as more regular than it physically is” (Eriksson, 1991:62). But what is critical for the rhythm class hypothesis is that it is not whether listeners hear something rhythmical in a language, but whether they can consistently determine if a language is syllable-timed, stress-timed or mora-timed. This has been directly checked in Miller (1984) in which both trained phoneticians and naïve listeners are asked to classify languages as either stress-timed or syllable-timed. Not only is there no clear evidence that people have this ability, but also the classification by naïve listeners deviate from the rhythm class hypothesis more than trained phoneticians who are biased by the knowledge of the hypothesis. White et al. (2012) investigated how language pairs were categorized by looking at utterances that only retained durational features. They found that English listeners could distinguish between not only English and Spanish (from different rhythm classes), but also between different accents of British English. White et al. (2016) found that infants were able to distinguish French and Spanish (from same rhythm classes). Arvaniti and Rodriquez (2013) found that discrimination was possible both across and within rhythm classes when speaking rates differed between context and test.

While the perception findings demonstrate that languages may not be neatly classified in the way predicted by rhythm class hypothesis, listeners’ sensitivity to rhythm nevertheless suggests that it may play a role in controlling timing and duration of speech. But it is also known that timing and duration are affected by factors with linguistic functions. These include, in particular, lexical stress (Fry, 1958; Klatt, 1976), boundary strength (Lehiste, 1972; Nakatani et al., 1981; Shattuck-Hufnagel and Turk, 1996; Xu and Wang, 2009), and intrinsic duration of segments (Klatt, 1976). For lexical stress, Fry (1955, 1958) has shown that vowel duration is a major correlate of lexical stress in both production and perception in English. The stressed/unstressed duration ratio can be as large as 2.18:1 in English (Crystal and House, 1988), for example. For boundary marking, a function to break up continuous speech into smaller chunks for the ease of comprehension (Lehiste, 1972; Cutler et al., 1997; Schafer et al., 2000; Xu, 2019), a major timing cue is pre-boundary lengthening, i.e., elongation of the syllables and their component segments before a prosodic boundary (Lehiste, 1972; Nakatani et al., 1981; Shattuck-Hufnagel and Turk, 1996; Xu and Wang, 2009). Intrinsic duration of segments is defined as the relative duration of vowel and consonant regardless of other factors (Peterson and Lehiste, 1960; Klatt, 1976), and it is measured as the average duration of each segment (van Santen and Shih, 2000). The presence of the above-mentioned duration-affecting factors means that genuinely independent rhythmic effects must be above and beyond all the functional effects.

There has already been some research in this direction, although not always with the goal to search for evidence of a tendency toward isochrony. A question we examine in this paper is to what degree syllable duration is a function of the intrinsic duration of the segmental make-up (i.e., consonants and vowels) of the syllable. There are multiple findings that syllable duration in English increases quasi-linearly with syllable size, i.e., the number of constituent segments (O’Connor, 1968; Crystal and House, 1990; van Santen and Shih, 2000). Based on the database they examined, van Santen and Shih (2000) showed that syllable duration is highly predictable from segmental duration in English, i.e., with every increment in the intrinsic duration of segments, syllable duration increases by almost the same amount. One interpretation of this finding is that English syllable duration is not flexible enough to allow for any purely rhythm-driven timing control in the language. Interestingly, however, the authors found, in the same study, that syllable duration in Mandarin is not as highly correlated with vowel duration as in English. Our interpretation, not contemplated in van Santen and Shih (2000), is that Mandarin syllable duration is more flexible than that of English, such that in Mandarin, syllable duration can indeed be described as showing a tendency toward rhythmic timing. We note, however, that the data is from one male speaker for English and one male speaker for Mandarin in their study, and thus, the generalizability of their findings is not yet clear.

Along these lines, Nakatani et al. (1981) found the duration of inter-stress intervals in English is at least linearly related to the number of constituent syllables, and that there is actually some accelerated increase of the interval duration with interval size. In that study, reiterant speech, whereby all syllables were replaced by [ma], was used to eliminate the segmental effects, which may have reduced the relevance of the findings to fully natural speech. But a similar linear relationship between the number of intervening unstressed syllables and the inter-stress interval for real words in sentence context was also found by Lea (1975) for English, although it was reported only in a conference abstract. These findings further suggest that English syllables are probably not compressed to maintain equal inter-stress intervals as the size of the inter-stress interval increases.

Nakatani et al. (1981) also examined how syllable duration is affected by lexical stress and position in word and phrase. They found that both lexical stress and word/phrase position have clear effects on syllable duration, but the two kinds of effects work in parallel. For the positional effect, word-initial syllables are slightly longer than word-medial syllables, and interestingly, the duration of word-final syllables are roughly the same independent of whether the word is monosyllabic or multi-syllabic. However, they did not provide statistical reports on these effects. Xu and Wang (2009) found in Mandarin that phrase-medial syllables are shorter than phrase-initial syllables, and phrase-final syllables in multi-syllabic phrases are shorter than mono-syllabic words. Yuan and Liberman (2015) reported that word-medial plosives and affricates are more likely to be reduced than word-initial ones, which can be interpreted as a sign of shorter word-medial syllables than word-initial syllables. Compared to English, Mandarin therefore may have two additional means to shorten phrases as their sizes increase. One is to shorten phrase-medial syllables compared with phrase-initial syllables and the other is to shorten phrase final syllables from multisyllabic phrases compared with monosyllabic phrases. This makes it likely that Mandarin has a tendency toward equal duration of phrases, which, by the way, would run counter to the widely held belief that Mandarin is syllable-timed based on auditory impression and traditional analyses (Lin and Wang, 2007) as well as rhythm metrics (Grabe and Low, 2002; Lin and Wang, 2007; Mok and Dellwo, 2008; Nolan and Asu, 2009).

In addition to intrinsic duration of segments and lexical stress, another important factor affecting timing and duration is boundary strength. Continuous speech is known to be broken up into smaller chunks, both for ease of perceptual comprehension and for production. Of the variety of cues that have been reported, two are of particular importance, namely, pre-boundary lengthening and silent pause (Lehiste, 1972; Xu, 2009). The amount of pre-boundary lengthening is related to the strength of the boundary: the greater the strength, the longer the duration (Nakatani et al., 1981; Fougeron and Keating, 1997; Xu and Wang, 2009). Silent pause, the second important boundary cue, is often associated with a strong boundary (O'Malley et al., 1973; Lea, 1980; Swerts, 1997; Wang et al., 2019).

For pre-boundary lengthening, interestingly, there is already some evidence of cross-language differences. For English. Wightman et al. (1992) showed significantly different amounts of pre-boundary lengthening among all four levels of boundary strength: prosodic word, a group of words within a larger unit, intermediate phrase, and intonational phrase. For Mandarin, Yang (1997) reported that syllable duration increases before word group and phrases boundaries, but then decreases before clause and sentence boundaries. This is partially corroborated by Li (1998) and Yang and Wang (2002), who found no significant difference in pre-boundary lengthening between minor prosodic phrase and major prosodic phrase boundaries.

For silent pause, Wightman et al. (1992) showed that in English, unfilled pauses occurred in 23% of the “intonation phrase” boundaries while for “groups of intonation phrases,” 67% had unfilled pauses. For Mandarin, normally there is no silent pause following a prosodic word, but as boundary strength increases so does silence duration (Qian et al., 2001; Yang and Wang, 2002; Xiong, 2003; Wang et al., 2019). This seems to suggest a trading relation between pre-boundary lengthening and silent pause for larger boundaries in Mandarin, which is consistent with suggestions that cues of lengthening and pausing may counterbalance each other (Lehiste, 1979; Scott, 1982). Pre-boundary lengthening and silent pause may be seen to combine to form a joint boundary strength cue, as both affect the temporal distance between the onsets of the pre-boundary constituent and the post-boundary constituent (Xu and Wang, 2009). This has seen some initial support from an empirical study (Wang et al., 2018).

Given the duration–affecting linguistic factors, if rhythm is indeed an additional timing factor, its effects should be detectable in the form of a tendency toward isochrony when the major linguistic factors are controlled for. The present study is a corpus analysis with a two-fold goal: (1) to find out if there is any tendency toward equal duration at either the syllable or the phrase level in English and Mandarin, and (2) to compare the two languages in terms of how they mark boundaries of different levels. The reason for a corpus study is, first, to allow us to examine previous findings from controlled experiments in a more naturalistic setting, as the corpora used were not designed for experimental purposes. Second, it would allow us to examine break levels that are higher than those in most previous investigations. More specifically, the following questions are examined:

1. Is there an isochrony tendency in English after controlling for stress and break level? More specifically:
  - Are English segments adjustable toward equal syllable duration?
  - Are English syllables adjustable toward equal inter-stress interval duration?
  - Are English syllables adjustable toward equal phrase duration?
2. Is there any duration compression in Mandarin after controlling for break level?
  - Are Mandarin segments adjustable toward equal syllable duration?
  - Are Mandarin syllables adjustable toward equal phrase duration?
3. Are there differences between English and Mandarin in terms of pre-boundary lengthening?
4. Are there differences between English and Mandarin in marking high-level boundaries?

For 1a and 1b, we corroborate previous findings of linear relation between segment duration and syllable duration (O’Connor, 1968; Crystal and House, 1990; van Santen and Shih, 2000) in English, and between syllable duration and duration of interstress intervals in English (Shen and Peterson, 1962; Bolinger, 1965; O'Connor, 1965; Lea, 1974; Nakatani et al., 1981). For 1c, we examine whether there is a linear relation between syllable duration and phrase duration, where phrases may or may not coincide with interstress intervals. For 2a, we ascertain whether there is a linear relation between segment duration and syllable duration in Mandarin, just as in English, or segments are somewhat compressible to make syllables equally long. Previous findings on this, as mentioned above, have been equivocal (van Santen and Shih, 2000). For 2b, we will try to find out if in Mandarin, unlike in English, syllable duration is compressible to make it possible to approach equal duration of phrases. Previous findings by Xu and Wang (2009) have shown indications that this may be possible, as mentioned earlier. For (3), we examine how pre-boundary lengthening differentiates break indices (i.e., boundary strengths) in both languages. This is to confirm results from previous studies (Wightman et al., 1992; Yang, 1997; Li, 1998; Yang and Wang, 2002; Cao, 2005). Finally, for (4) we test the hypothesis that pre-boundary lengthening and silent pause can be combined to indicate a relational distance between adjacent constituents (Xu and Wang, 2009).

## 2. Materials and methods

### 2.1. English corpus

For English, the Boston University Radio News Corpus was used (Ostendorf et al., 1995). It consists of news stories recorded by three female and four male FM radio news announcers during broadcast and the same four type-B news stories recorded by six of the seven announcers in a laboratory condition. Professional radio announcers tend to be more fluent than non-professional speakers, producing fewer disfluencies and prosodic errors (Ostendorf et al., 1995). The overall speech rate is 5.31 syllables per second. The paragraphs are annotated previously with orthographic transcriptions, phonetic alignments, part-of-speech tags and prosodic labels in the ToBI system (Ostendorf et al., 1995). The ToBI (tone and break indices) system marks prosodic phrasing, phrasal prominence and boundary tones. For lexical stress, only two levels are distinguished: stressed and unstressed. The phonetic alignments are generated automatically using constrained speech recognition (Kimball et al., 1992). Segmentation times and phone durations are provided in units of 10 milliseconds. Annotation for the news recorded in the laboratory were hand-corrected by the corpus developer, while those recorded during broadcast were not. In our analysis, data from one of the male speakers were excluded for not having prosodic information. All other announcers’ data with enough segment, syllable, and prosodic information were used. The amount of data analyzed is therefore greater than in other studies that also made used of this corpus (Sun, 2002; Choi et al., 2005).

One problem with the corpus was that words were divided into syllables based on a dictionary that combined MOBY and SRI dictionaries, which did not consider resyllabification (Kelso et al., 1986; de Jong, 2001; Gao and Xu, 2010). For example, the dictionary divided the word *decade* into “d eh + 1 k” and “ey d.” In spoken English, speakers tend to say it as “d eh + 1” and “k ey d,” so that “k” is an onset. Resyllabification was therefore performed based on the following rules: (1) within a word, if a coda is followed by a syllable beginning with a vowel, the coda is treated as the onset of the next syllable (Campbell and Isard, 1991); (2) between words, if a coda is followed by a syllable beginning with a vowel, and the break index (Beckman and Ayers, 1997) is 1 or 2 without silence, the coda is also treated as the onset of the next syllable.

### 2.2. Mandarin corpus

The Mandarin data were from Annotated Speech Corpus of Mandarin Discourse (ASCCD, Li et al., 2000b), which was set up and recorded at the Institute of Linguistics, Chinese Academy of Social Sciences. There are 18 discourses, each consisting of 300–500 syllables and several paragraphs. Five male and five female Beijing speakers who speak standard Mandarin read aloud the discourses naturally (Li et al., 2000a). Some of the speakers are teachers with a Phonetics background. The overall speech rate is 5.16 syllables per second. Four annotation tiers, including the syllable tier, initial and final (onset and rhyme) tier, break index tier and stress tier, were labeled (Li et al., 2000a). In total, 41,673 CV syllables, 18,486 CVC syllables and 10,647 CGV syllables were analyzed. The CGV structure is unique to Chinese, where G stands for the semivowel glide between onset and nucleus.

An advantage of both corpora is that they are already annotated with break index by the developers. This provides a level of objectivity in our data analysis, although the definitions of the break indices are not identical for the two languages, as will be explained.

### 2.3. Measurement

#### 2.3.1. Syllable duration related to segments

To understand whether segments are compressed if their intrinsic duration is relatively long, we examined how closely syllable duration is correlated with the intrinsic duration of segments (estimated average duration), similar to what is investigated by van Santen and Shih (2000). To make our results comparable, we made our measurements as similar to theirs as possible.

Suppose we analyze CV syllables that share the same context, with the same stress, the same structure in terms of number of segments and their order. Then the only difference between these syllables is their segmental makeup such as whether a syllable starts with a [t] or a [b]. Likewise, for CV syllables starting with the same consonant, the only difference would be whether the vowel is, e.g., [u] or [i]. van Santen and Shih (2000) have shown that, under these circumstances, syllable duration is highly predictable from segmental duration in English. Interestingly, however, the data in the same study showed that in Mandarin, syllable duration is not as highly correlated with vowel duration as in English. This language difference, however, is not elaborated in van Santen and Shih (2000).

For syllables of the type CV, DUR(*c*•) is the mean duration of all CV syllables starting with *c*; DUR(*c*|*c*•) is the duration of c averaged over all vowels; and D_inherent_(*v*) is the inherent duration of a vowel. This method also works for vowels.


(1) $$DUR\left(c\cdot \right)=\alpha DUR\left(c|c\cdot \right)+\left(1/V\right)\overset{v=V}{\underset{v=1}{\sum }}D_{inherent}\left(v\right)+\beta$$

**Figure 1:**
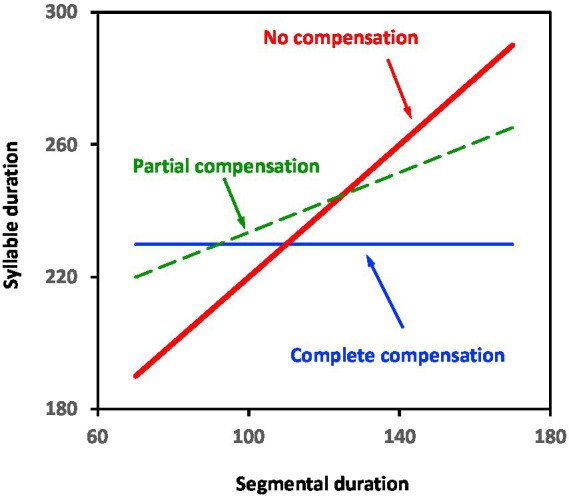
Schematic drawing of relation between syllable and segmental duration with complete, partial, or no compensation, where “compensation” refers to how much the duration of a consonant or vowel depends on the identities of the remaining segments in the syllable (adapted from van Santen and Shih, 2000).

Equation (1) shows the compensation effect in a syllable, as it measures how much the duration of a consonant or vowel depends on the identities of the remaining segments in the syllable. The duration of the syllable as a function of segmental duration is illustrated in Figure 1, where *α* represents the slope of the regression line. When *α* is 1, there is no compensation. When *α* is 0, there is complete compensation. Values of *α* between 0 and 1 indicate that there is partial compensation, hence, partial compression and/or elongation of segments in the direction of making syllables equally long.

Our investigation differs from van Santen and Shih (2000) in two ways, however. Firstly, they used an English database consisting of 2017 isolated sentences read by one American English male speaker and a subset of a database consisting of 424 Mandarin sentences recorded by one male Mandarin speaker. We used 369 paragraphs of news in English from three female speakers and three male speakers and a Mandarin corpus consisting of 18 discourses spoken by 10 speakers. Secondly, they reported only results of stressed word-initial CV syllables in phrase-medial words and stressed word-final CVC syllables in accented phrase-medial words in English, without considering consonant clusters. We treated consonant clusters as singletons and included CV and CVC syllables in all positions. More detailed differences are shown in Table 1.

**Table 1:** Differences between van Santen and Shih’s, 2000 and current study.

		van Santen and Shih, 2000	Current study
Corpus	English	One male speaker 2017 isolated sentences	Three female and three male speakers 369 paragraphs of news
	Mandarin	One male speaker One subset of a database consists of 424 Mandarin sentences	Five female and five male speakers 18 discourses
Syllable	English	CV and CVC Stressed syllables only No consonant clusters Certain positions	CV and CVC (C here includes both consonants and consonant clusters) All syllables Consonant clusters All positions
	Mandarin	Certain positions	All positions

The underlying assumption is that the control of the effects of linguistic factors, i.e., lexical stress and boundary strength, is accomplished by using a very large number of syllables across all conditions to even out the influence, which may be compared to the use of long-term spectrum to examine speaker characteristics (Hollien and Majewski, 1977). An important reason for applying this control method is that intrinsic duration and syllable duration are both affected by stress and break index, and it would be difficult and unnecessary to separate their effects for the current purpose. Future research is needed to investigate this assumption more carefully.

#### 2.3.2. Syllable duration in phrases

We also examined whether and how closely syllable duration is related to linguistic factors of stress, and position in words/phrases; also, how inter-stress interval duration is related to number of syllables. Here our method is similar to that of Nakatani et al. (1981), but with three major differences as shown in Table 2.

**Table 2:** Differences between Nakatani et al. (1981) and current study.

Nakatani et al. (1981)	Current study
Reiterant speech	Corpora of natural speech
Isolated sentences	Paragraphs and discourses
American English only	American English and Mandarin

## 3. Analysis and results

### 3.1. Compressibility of segments

For syllables to show a tendency toward equal duration, their component segments must exhibit compressibility in one of two ways, or both. First, a segment would be compressed if its intrinsic duration is relatively long, so as to better match the intrinsically shorter ones. Second, all segments would be compressed as the number of segments increases in a syllable. In the following, we will examine both kinds of compressibility.

#### 3.1.1. Relation of syllable duration to intrinsic segment duration

**Figure 2:**
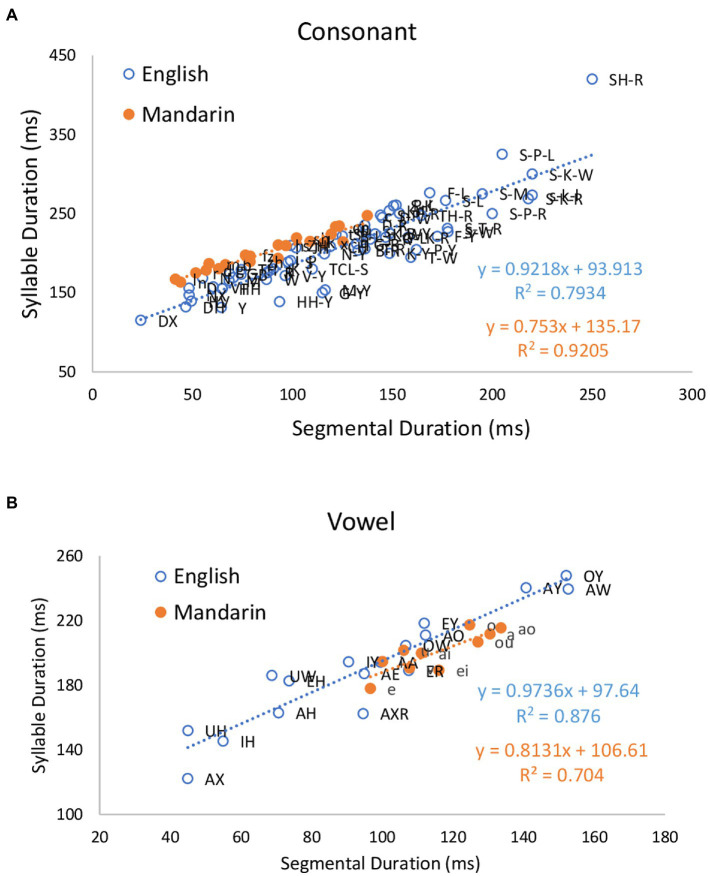
Duration of CV syllable in American English and Mandarin as a function of intrinsic duration of consonants **(A)** and vowels **(B)**, together with linear regression lines and Pearson correlation coefficients.

In this section, first we compare relation of CV syllable durations to intrinsic segment durations in American English and Mandarin. Figure 2 shows plots of syllable duration as a function of intrinsic durations of onset and nucleus segments in CV syllables in American English (*N* = 18,941) and Mandarin (*N* = 41,673), and coefficients of Pearson correlation coefficients. For English, the coefficients are 0.891 (*p* < 0.001) and 0.936 (*p* < 0.001), and the slopes of regression lines are 0.9218 and 0.9736, respectively. For Mandarin, the Pearson correlation coefficients are 0.959 (*p* < 0.001) and 0.839 (*p* < 0.001), and the slopes of regression lines are 0.753 and 0.8131, respectively. In both languages, therefore, syllable duration is closely related to the intrinsic durations of the onset and the nucleus, but the slopes of regression lines are shallower in Mandarin than in English for both consonants and vowels.

**Figure 3:**
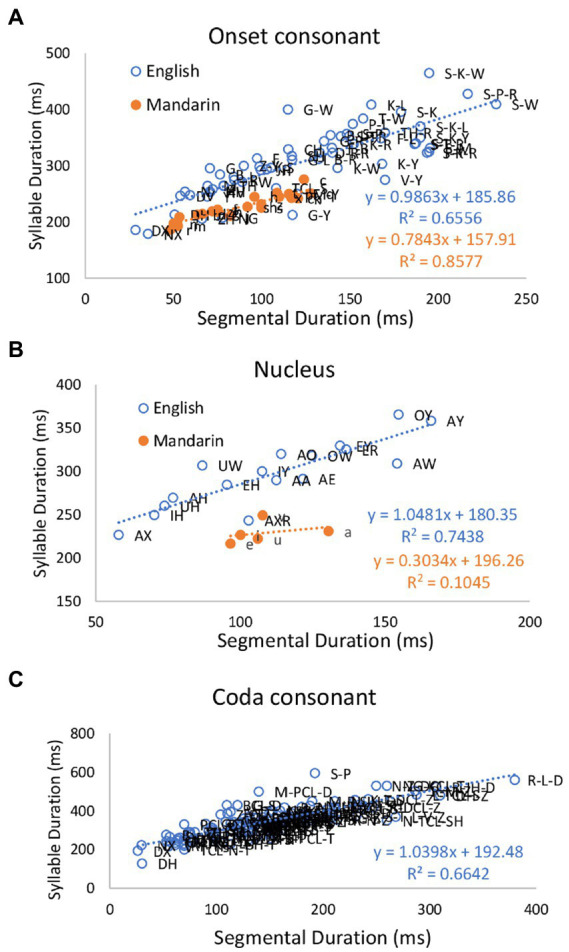
Effects of consonant **(A)** and **(C)** and vowel **(B)** identity on CVC syllable duration in American English and Mandarin.

Next, we compare relation of CVC syllable durations to intrinsic segment durations in American English with that in Mandarin. Codas are not analyzed in Mandarin, because there are only two codas, /n/ and /ŋ/, and they were not segmented in the corpus, so it was impossible to get their intrinsic durations. Figure 3 shows plots of syllable duration as a function of intrinsic durations of onset, nucleus and coda segments in CVC syllables in American English (*N* = 17,354) and Mandarin (*N* = 18,486), and Pearson correlation coefficients. For English, the correlations between syllable durations and segmental durations are 0.810 (*p* < 0.001) for the onset consonant, 0.862 (*p* < 0.001) for the vowel, and 0.815 (*p* < 0.001) for the coda consonant, and the slopes of the regression lines are 0.9863, 1.0481, and 1.0398, respectively. For Mandarin, the correlations between syllable duration and segmental durations are 0.926 (*p* < 0.001) for the onset, 0.323 for the vowel, and the slopes of regression lines are 0.7843 and 0.3034, respectively.

**Figure 4:**
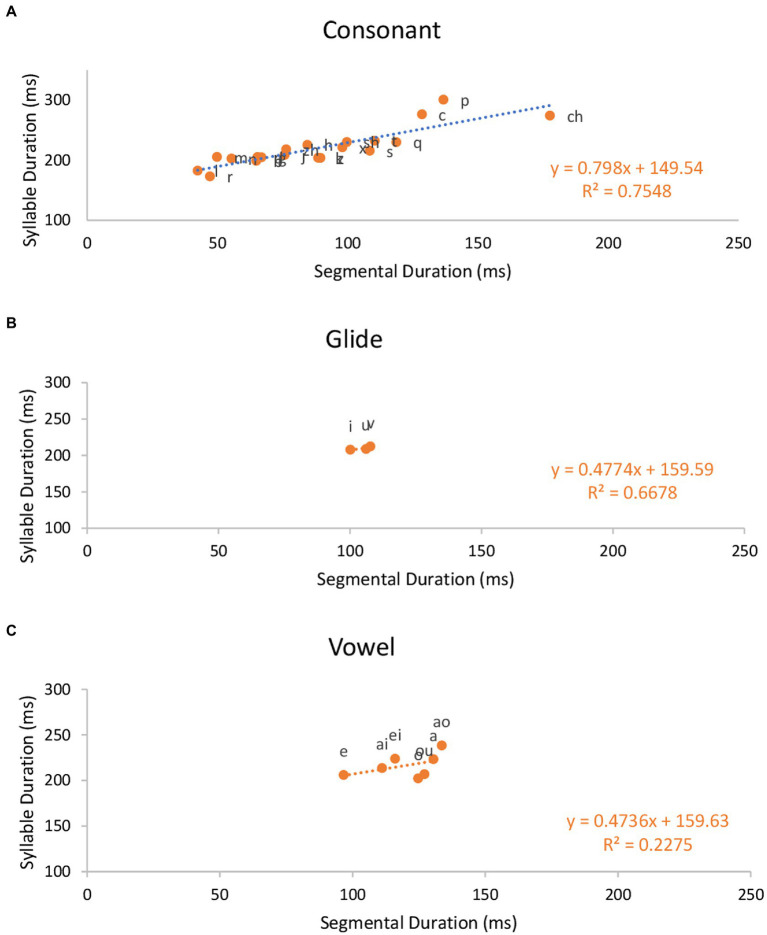
Effects of onset consonant **(A)**, glide **(B)** and vowel **(C)** identity on CGV syllable duration for Mandarin.

Next, we focus on CGV syllables in Mandarin, where G indicates a glide. Figure 4 shows plots of syllable duration as a function of intrinsic durations of consonant, glide and vowel in CGV syllables in Mandarin (*N* = 10,647), and Pearson correlation coefficients. The correlations between syllable duration and segmental durations are 0.869 (*p* < 0.001) for the onset consonants, 0.817 for the glides (*p* < 0.001) and 0.477 for the vowels (*p* = 0.279). The slopes of the regression lines are 0.798, 0.4774, and 0.4736, respectively.

The most important result shown so far is that the slopes of the regression lines for syllable duration as a function of intrinsic duration of segments are close to 1 for English in both CV and CVC syllables. These results can be interpreted as showing that compared to Mandarin, English segments maintain their intrinsic durations; the segments are neither compressed nor stretched to make syllables equal in duration. In contrast, for Mandarin, the slopes of the regression lines are well below 1.0 in both CV and CVC syllables, especially in the latter. The slopes are especially shallow for vowels and glides. This suggests that Mandarin has a tendency to adjust segment duration in order to maintain a constant syllable duration.

#### 3.1.2. Relation of syllable duration to syllable size

**Figure 5:**
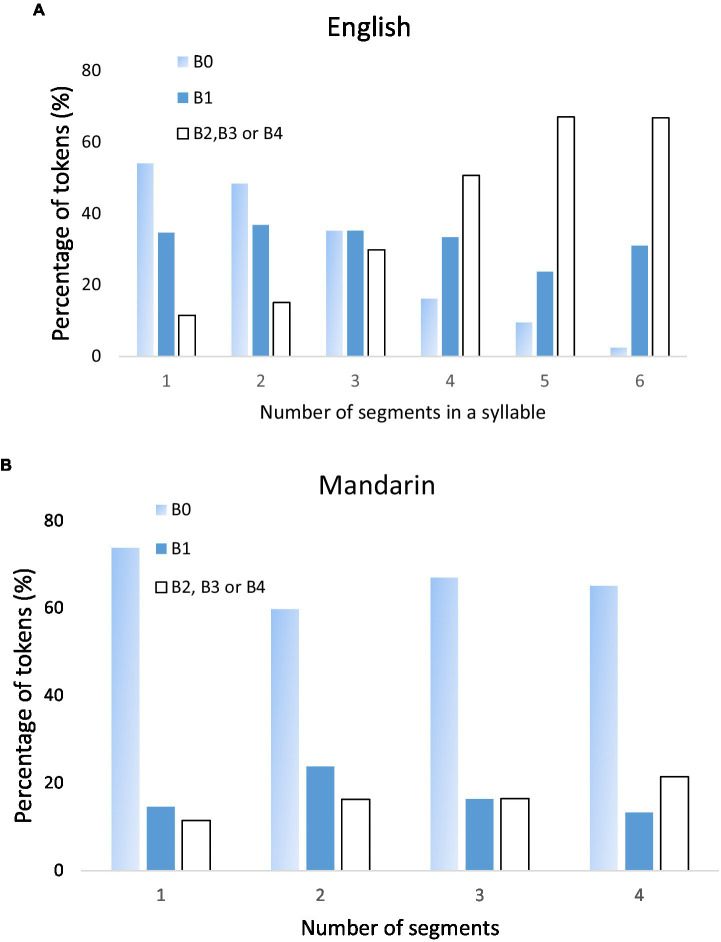
Histograms of distribution (% of tokens) of syllables of different sizes before different levels of boundaries (*x*-axis) in English **(A)** and Mandarin **(B)**.

In this section, we examine only the syllables that occur before a B1 boundary. A B1 boundary refers to most phrase-medial word boundaries in English (Beckman and Ayers, 1997) and prosodic word boundary (Li, 2002); Hence, these syllables never occur in final phrase/final utterance position. For the relation of syllable duration and syllable size (number of component segments), a potential confounding factor is that, in English, there is an uneven distribution of syllables of different sizes across boundaries of various strengths. Figure 5 shows histograms of syllables of various sizes at different boundary indices. As can be seen, 53.98% of the one-segment syllables occur before B0, while 66.67% of the six-segment syllables occur before a phrase boundary (B2, B3, and B4). In contrast, syllables of different sizes are much more evenly distributed before B1. Although the same trend is not seen in Mandarin, to avoid the potential bias, in the following analysis, we thus include only syllables before B1 in both English and Mandarin. Also excluded from the analysis are syllables with the neutral tone in Mandarin. In total 10,478 Mandarin syllables and 15,161 English syllables were included in the analysis.

**Figure 6:**
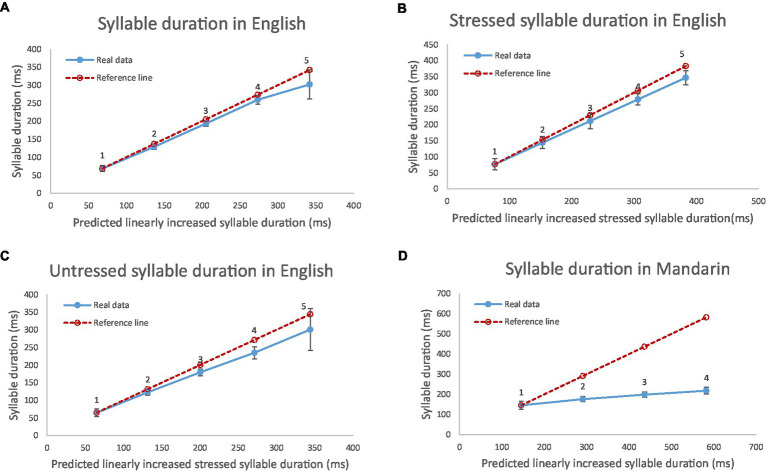
Mean syllable duration in English and Mandarin as a function of syllable size (number of component segments) for all English syllables **(A)**, English stressed syllables **(B)** English unstressed syllables **(C)** and Mandarin syllables **(D)**. In each plot the dashed line is the reference line consisting of points each with the same increment as the duration of monosyllabic words (leftmost point). The number of syllables is indicated by a number above each point in the graph.

Figure 6 shows syllable duration in English and Mandarin as compared to the linear reference (dashed) lines. Because stress plays a role in the relation between syllable size and syllable duration, especially in English, the results from stressed and unstressed syllables are presented separately. As can be seen, as the number of segments increases, syllable duration increases almost linearly in English, although the rate of increase is reduced slightly in the most complex syllables (those consisting of five segments). In Mandarin, in contrast, the rate is substantially reduced starting from 2-segment syllables.

**Figure 7:**
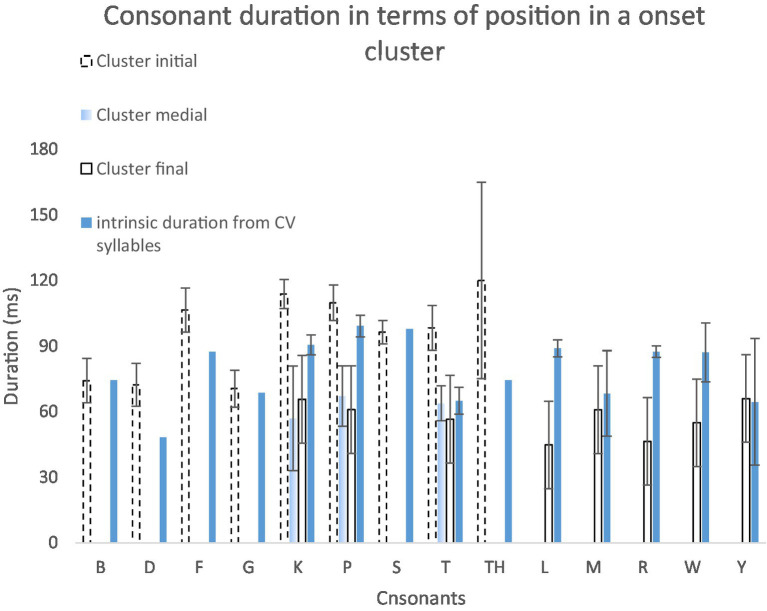
Consonant duration in initial, medial, final position in onset consonant clusters or as singletons, compared to the intrinsic durations of the same consonants calculated from CV syllables. CCCVC, CCCVCC, CCVC, CCVCC, CCVCCC syllables are pooled together.

**Figure 8:**
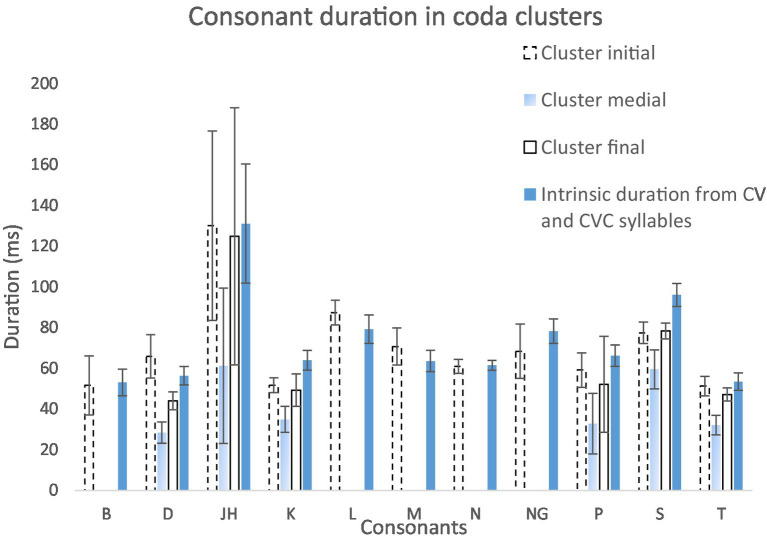
Consonant duration in initial, medial, final position in coda consonant clusters and intrinsic durations of the same consonants in coda, calculated from CVC syllables. CCCVCC, CCVCC, CCVCCC, CVCC, CVCCC syllables are pooled together here.

The reduction of rate of increase in syllable duration as a function of syllable size in English (Figures 6A–C) occurs only in syllables consisting of five segments. The source of this reduction is likely consonant clusters, as shown in Figures 7, 8. These two figures display durations of each consonant in different locations in a cluster or as a singleton as compared to its intrinsic duration from CV syllables.

Consonant duration at different within-cluster locations were compared with their intrinsic duration by Paired samples *T*-tests. Initial consonants (*M* = 9.57, SD = 1.89) are significantly longer than their intrinsic durations (*M* = 7.84, SD = 1.68); *t*(8) = −3.227, *p* = 0.012, *n* = 9, while final consonants (*M* = 5.71, SD = 0.80) are significantly shorter than their intrinsic durations (*M* = 8.14, SD = 1.34); *t*(7) = 3.954, *p* = 0.006, *n* = 8. Although there is a trend that medial consonants (*M* = 6.27, SD = 0.52) are shorter than their intrinsic durations (*M* = 8.49, SD = 1.78), there is no statistical significance; *t*(2) = 2.108, *p* = 0.170, *n* = 3.

Figure 8 shows intrinsic consonant duration and their duration in different locations within a coda consonant cluster. Paired samples *T*-tests show that there is no significant difference between initial consonants (*M* = 7.04, SD = 2.28) and their intrinsic durations (*M* = 7.30, SD = 2.32); *t*(10) = 0.958, *p* = 0.361, *n* = 11. Medial consonants (*M* = 4.15, SD = 1.48) are significantly shorter than their intrinsic durations (*M* = 7.78, SD = 3.01); *t*(5) = 5.171, *p* = 0.004, *n* = 6. Final consonant (*M* = 6.60, SD = 3.14) are also significantly shorter than their intrinsic durations (*M* = 7.78, SD = 3.01); *t*(5) = 6.227, *p* = 0.002, *n* = 6.

To summarize, despite a lengthening effect on initial consonants in onset clusters, there are significant shortening effects on final consonants in onsets, and on medial and final consonants in codas. Compression of consonant clusters may therefore be a main source of shortening in syllables consisting of five or more segments in English.

### 3.2. Compressibility of syllables

The results reported in Section 3.1 show that English segments are not compressible for the sake of equal syllable duration, which contrasts with Mandarin where segments are clearly compressible in the direction of making syllables of different sizes equally long. This seems to be consistent with the classification of Mandarin as a syllable-timed language and English as a non-syllable-timed language. But it also leaves open whether either of the two languages shows a tendency toward equal timing at a level above the syllable. In the following analyses, we will examine for English if there is any tendency toward equal inter-stress intervals, and for Mandarin if there is any tendency toward equal phrase duration.

#### 3.2.1. Inter-stress intervals in English

According to the Rhythm Class Hypothesis, inter-stress intervals are constant in a stress-timed language. If this is true, inter-stress intervals should maintain a constant duration regardless of the number of syllables in an interval, or at least show a tendency in that direction. Inter-stress intervals that were not phrase-final and immediately followed by a stress were included in the analysis (Nakatani et al., 1981). To assess the relationship between the number of syllables and inter-stress interval duration, we measured from the onset of a stressed syllable to the onset of the next stressed syllable. We only considered intervals with one to four syllables, because each have more than 30 tokens from each speaker in our data. Longer intervals have too few tokens to guarantee reliability. In total, 7,184 intervals were analyzed.

**Figure 9:**
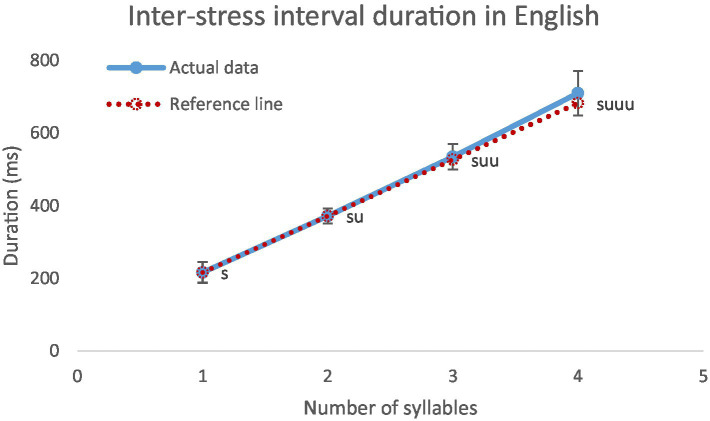
Average inter-stress interval durations in seconds, s indicates a stressed syllable and u indicates an unstressed syllable. On the reference line, the leftmost point shows the duration of monosyllabic stressed syllable, while the rest of the points shows the same increment of the unstressed syllable in disyllabic intervals.

Figure 9 shows the average duration of inter-stress intervals as a function of size in terms of number of constituent syllables. Inter-stress interval duration is highly related to interval size. The correlation between inter-stress interval duration and interval size is 0.981 (*p* < 0.001). Every unstressed syllable added increased inter-stress interval duration by 155 ms. This is consistent with previous findings (Classe, 1939; Shen and Peterson, 1962; Bolinger, 1965; O'Connor, 1965; Lea, 1974). Contrary to the prediction of English being a stress-timed language that the inter-stress intervals are constant regardless of number of syllables, the inter-stress intervals are linearly related to the number of syllables. In fact, it is somewhat positively accelerated. That is, as the interval size increases, there is a tendency for the increase in interval duration to accelerate. Similar acceleration has also been found by Nakatani et al. (1981), although they excluded intervals with inter-stress function words. Speculatively, as the size increases, a stress group is more and more likely to break up into sub-intervals, and the boundary of the sub-intervals are marked by final lengthening, which would in turn increase the duration of the inter-stress interval as a whole. This possibility has also been raised by Uldall (1971, 1978). But systematic studies are needed in the future to examine it in depth.

#### 3.2.2. Compressibility of syllables in prosodic phrases in Mandarin and English

Mandarin does not have lexical stress that is equivalent to word stress in English. Even though the neutral tone shows phonetic properties similar to those of English unstressed syllables (Xu and Xu, 2005; Chen and Xu, 2006), its occurrence is infrequent. This makes it impossible to compare inter-stress intervals between the two languages. The two languages can be compared, however, in terms of phrase duration. This section examines for English and Mandarin whether syllables are compressed as the number of syllables in a phrase increases. Here we extracted prosodic phrases based on the break indices at or above B2 in the two corpora, i.e., B2, B3, and B4. In the English corpus, B2 refers to a lower-level perceived grouping of words that does not have an intermediate B3 or a B4 intonational boundary marker or the disjuncture with next word is weaker than expected although the pitch pattern clearly suggests an intermediate B3 or a B4 intonation phrase boundary (see Beckman and Ayers, 1997). In Mandarin, B2 refers to minor prosodic phrase boundary; B3 refers to major prosodic phrase boundary; and B4 refers to prosodic group boundary (Li, 2002). Due to the difference in definitions, therefore, the durational relation of syllable to phrase can be compared only in terms of trends.

For English, lexical stress is a confounding factor, because each word can have only one primary stress and stressed syllables are much longer than unstressed syllables (Nakatani et al., 1981; Crystal and House, 1988). Thus, an increase in word length is necessarily achieved by adding more unstressed syllables, which may generate the appearance of a tendency toward equal duration of stress groups. To control for stress, the duration difference of each segment or consonant cluster was calculated and added to every segment and consonant cluster in an unstressed syllable when computing phrase duration.

The first step was to calculate the differences between stressed segments and unstressed segments. Take [ei] as an example, stressed [ei] duration was calculated as an average duration of [ei] from all CV stressed syllables ending with [ei]. Unstressed [ei] duration was calculated as an average duration of [ei] from all CV unstressed syllables ending with [ei]. The difference between averaged stressed [ei] duration and averaged unstressed [ei] duration is the vowel duration difference for [ei]. This method was applied to each vowel. Those vowels that do not have both stressed and unstressed data were given a default duration difference which is the average duration difference of all other vowels that have data in both conditions.

Consonants were divided into onset and coda, and consonant clusters were treated as singletons. Take [p] for example, onset [p] duration in stressed condition was calculated as an average duration of [p] from all CV stressed syllables starting with [p]. Onset [p] duration in unstressed condition was calculated as an average duration of [p] from all CV unstressed syllables starting with [p]. The duration difference between averaged [p] in stressed condition and in unstressed condition is the duration difference for [p]. This method was applied to each onset consonant. Those onset consonants that do not have data in both stressed and unstressed conditions were given a default duration difference which is the average duration difference of all other onset consonants (excluding consonant clusters). Those consonant clusters that do not have data in both stressed and unstressed conditions were given a default duration difference which is the average duration difference of all other onset consonant clusters that have the same number of segments. A similar method was applied to coda consonant, in which the only difference is that the calculation was done on CVC syllables. With this method, the shorter duration of unstressed syllables was not attributed to the reduction of phrase duration. For Mandarin, phrases with one or more neutral tone syllables were excluded from the analysis, because neutral tone syllables are much shorter than syllables with full tones (Chen and Xu, 2006), but they are infrequent in the corpus.

**Figure 10:**
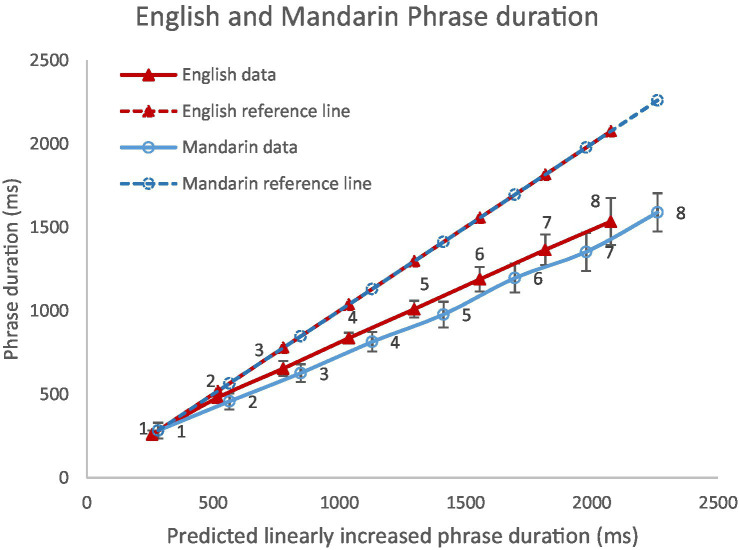
Average duration of phrases in English and Mandarin as a function of number of syllables in each phrase, in comparison with linearly predicted phrase duration.

Figure 10 shows phrase duration in English (*N* = 6,523) and Mandarin (*N* = 7,406) as a function of phrase size in comparison with predicted linearly increased phrase duration. The solid lines were drawn from phrases consisting of 1–8 syllables. The dashed lines (reference lines) refer to predicted phrase duration by treating monosyllabic phrase as the reference. As a result of these calculations, each additional syllable in the phrase is supposed to increase phrase duration by 259 ms in English and 283 ms in Mandarin if there is no tendency of equal duration. In both languages, phrase duration is strongly related to phrase size: Pearson correlation coefficients are 0.984 (*p* < 0.001) for Mandarin and 0.987 (*p* < 0.001) for English.

Compression can be seen in both languages from the graph. But this is likely because syllables in monosyllabic phrases, by definition, are phrase-final, which is subject to phrase-final lengthening. Using their mean duration as the baseline therefore provides an inflated reference slope, as phrase final lengthening does not apply to every syllable. To circumvent this problem, we then examined the compressibility of syllables as the number of syllables in a word increases. A word is defined as one that is marked by break index 1 in both the English and Mandarin corpora.

**Figure 11:**
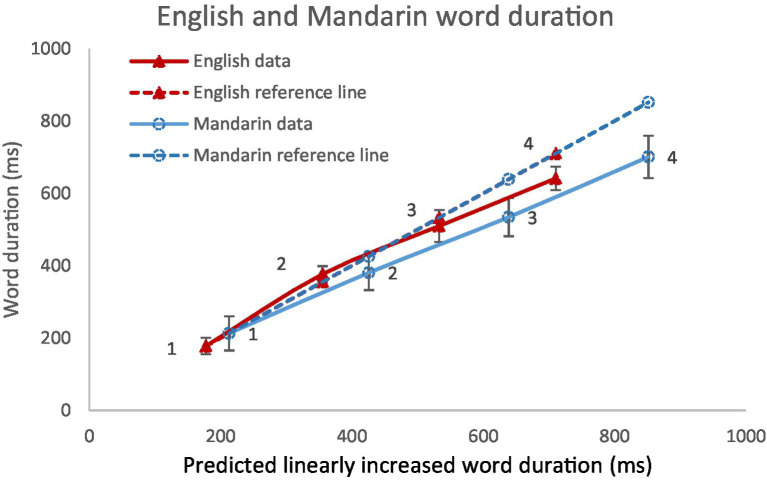
Word duration in English and Mandarin as a function of predicted linearly increased word duration.

Figure 11 shows word duration in English (*N* = 12,931) and Mandarin (*N* = 9,955) as a function of word size as compared with predicted linearly increased word duration. This time the reference lines are based on monosyllabic word duration. As can be seen, word duration is strongly related to word size in both language: Pearson correlation coefficients are 0.98 (*p* < 0.001) in Mandarin and 0.989 (*p* < 0.001) in English. But it can be also seen that syllables are compressed more in Mandarin than in English.

**Figure 12:**
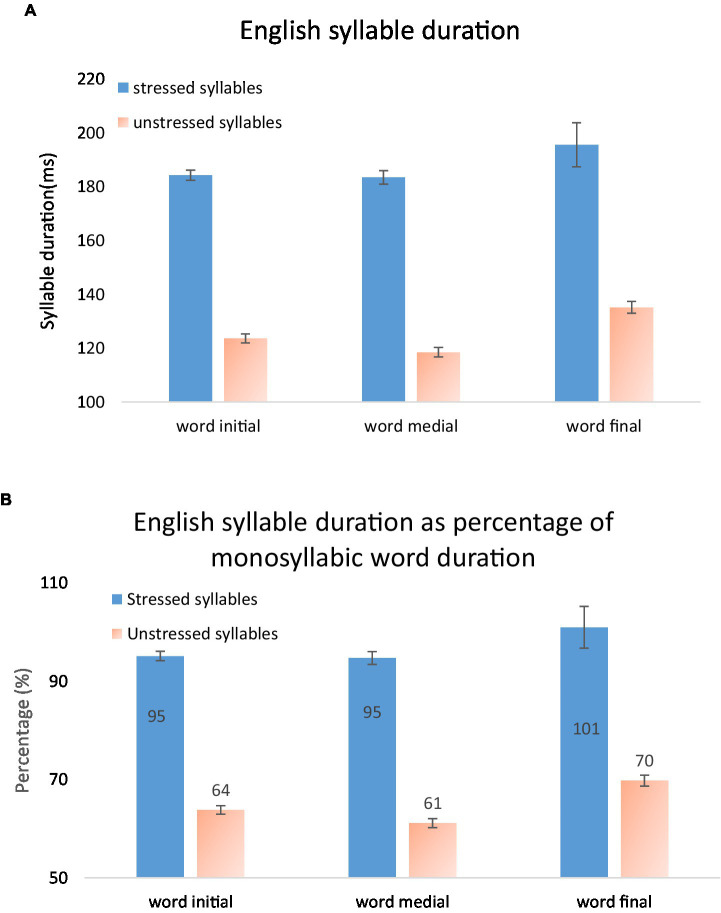
English syllable duration as a function of position in word, in **(A)** milliseconds, and **(B)** percentage of monosyllabic word duration.

To find out how syllables are compressed in words, we looked into syllable duration in terms of its position in word. In case the number of segments interacts with position, only CV syllables at word initial, medial and final positions were included in the analysis. In total 10,841 English and 13,571 Mandarin CV syllables were analyzed. Figure 12 shows how syllable duration depends on stress and position in word in English (A) or in terms of percentage of monosyllabic word duration (B). Note that when calculating monosyllabic word duration, highly frequent words like “to” are excluded from analysis. Word-final syllables are longer than word-initial and word-medial syllables; word-initial syllables are slightly longer than word-medial syllables, and monosyllabic words behave similarly as word-final stressed syllables.

Mixed Model ANOVAs were performed, with stress and position (word initial, word medial, word final) as fixed factors, subject as random factor and syllable duration as dependent variable. The results showed a main effect of stress: *F*(1, 5.123) = 159.150, *p* < 0.001, partial *η*^2^ = 0.969, and a main effect of position, *F*(2, 11.621) = 10.005, *p* = 0.003, partial *η*^2^ = 0.623. The effect of subject is not significant, *F*(5, 4.612) = 0.274, *p* = 908, partial *η*^2^ = 0.229. There were interactions between stress and subjects, *F*(5, 14.440) = 4.771, *p* = 0.009, partial *η*^2^ = 0.623, and between stress, position and subject, *F*(10, 10,805) = 3.591, *p* < 0.001, partial *η*^2^ = 0.03.

Bonferroni post-hoc analyses showed that word final syllables (*n* = 1878) are significantly longer than word initial (*n* = 5,040) and word medial syllables (*n* = 3,923). Although word initial syllables are slightly longer than medial syllables, they are not significantly different from each other. This may seem to differ from Nakatani et al.’s (1981) report that word-initial syllables were slightly but consistently longer than word-medial syllables. However, they did not support this observation with statistical analysis.

**Figure 13:**
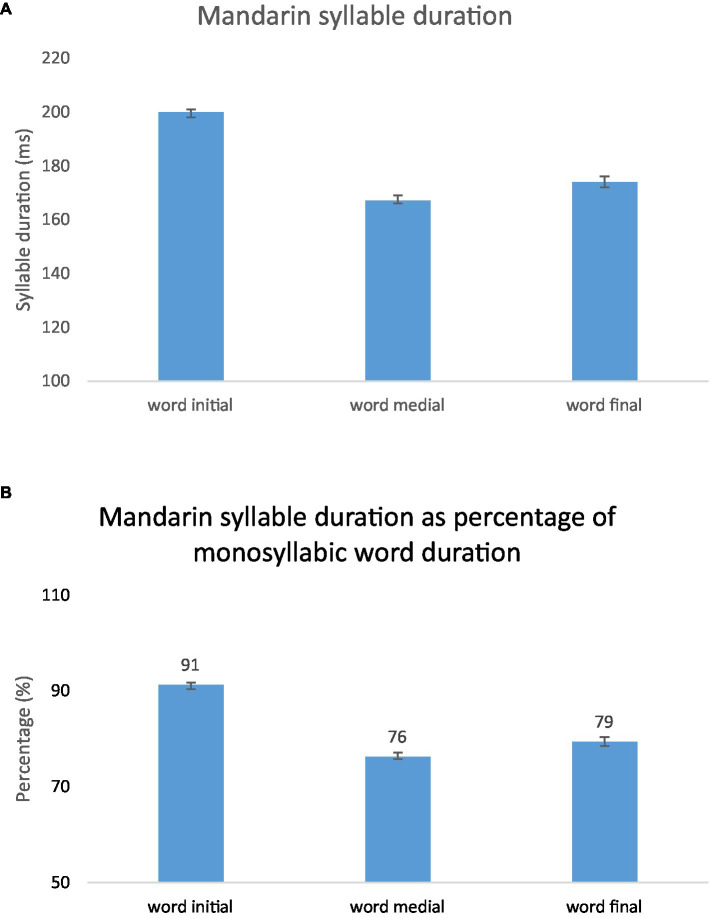
Mandarin syllable duration as a function of position in word, in **(A)** milliseconds, and **(B)** percentage of monosyllabic word duration.

Figure 13 shows Mandarin syllable duration as a function of position in word either in milliseconds (A) or in terms of percentage of monosyllabic word duration (B). Word-initial syllables are longer than word-medial and word-final syllables, and word-final syllables are longer than word-medial syllables. Mixed Model ANOVAs were performed on Mandarin data, with position (word initial, word medial, word final) as fixed factor, subject as random factor and syllable duration as dependent variable. The results showed a main effect of position, *F*(2, 21.101) = 160.133, *p* = 0.000, partial *η*^2^ = 0.938. The effect of subject is significant, *F*(9, 18.828) = 25.035, *p* = 0.000, partial *η*^2^ = 0.923. There was an interaction between position and subject, *F*(18, 15,701) = 5.224, *p* = 0.000, partial *η*^2^ = 0.006. Bonferroni post-hoc analyses showed significant difference on each pairwise comparison between positions (Word initial syllable *n* = 8,330, Word medial syllable *n* = 4,254, Word final syllable *n* = 3,147).

Comparing Figures 12A, 13B, we can see that syllable duration is more compressible in Mandarin than in English. First, In English, word-final syllables are about equally long as monosyllabic words, whereas in Mandarin word-final syllables are much shorter than monosyllabic words. Second, in English, word-initial syllables are slightly but not significantly longer than word-medial syllables. In Mandarin, in contrast, word initial syllables are much longer than word medial syllables. The combined effects of word medial shortening and word final shortening, therefore, make Mandarin words much more compressible in duration than English words, for which both effects are absent.

What is also interesting is that word-final syllables in Mandarin are not only shorter than monosyllabic words, but also shorter than word initial syllables. Compared with mono syllabic words, word initial-syllables are 9% shorter, while word-final syllables are 21% shorter. This means that there is no word-final lengthening in Mandarin.

### 3.3. Temporal cues for boundary marking

The lack of word-finding lengthening in Mandarin is also related to the third and fourth research questions of the study, namely, whether there are differences between English and Mandarin in terms of pre-boundary lengthening, and whether there are differences between the two languages in marking high-level boundaries. These questions will be answered by the results presented in this section.

#### 3.3.1. Mandarin results

**Figure 14:**
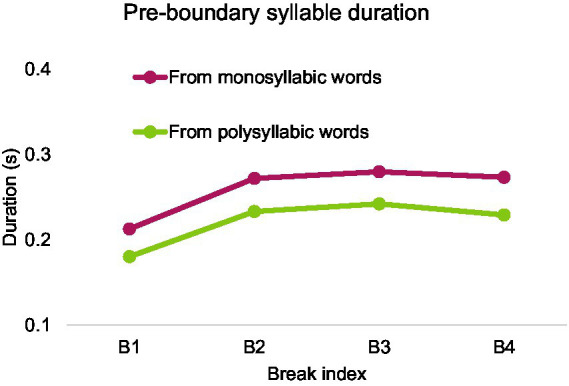
Pre-boundary syllable duration as a function of break index in Mandarin.

**Figure 15:**
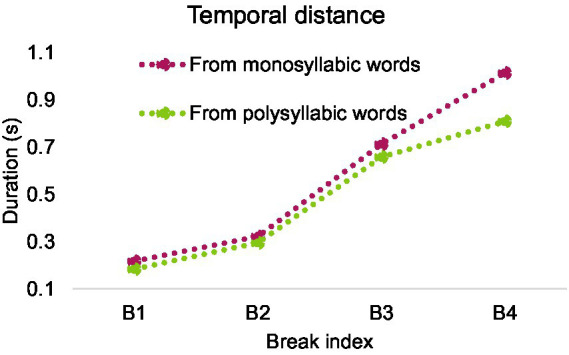
Temporal distance as a function of break index in Mandarin.

**Figure 16:**
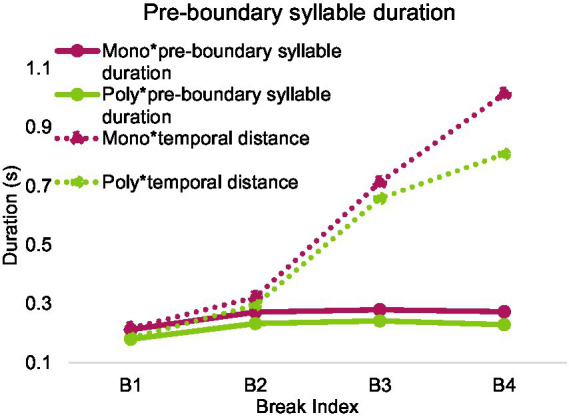
Pre-boundary syllable duration and temporal distance over break index in Mandarin.

After excluding syllables with neutral tones, 19,144 syllables from polysyllabic words and 2,777 syllables from monosyllabic words were included in analysis. Figures 14–16 show mean duration patterns in Mandarin. Figure 14 shows that pre-boundary syllable duration ceases to lengthen beyond break index 2. In contrast, as shown in Figure 15, temporal distance, which combines silent pause and pre-boundary duration, continues to increase beyond break index 2 for both monosyllabic and polysyllabic words.

A two-way repeated measures ANOVA was conducted, with the number of syllables (1 or more) in pre-boundary words and break index (1, 2, 3, and 4) as fixed factors, pre-boundary syllable duration as the dependent variable. The analysis was performed after calculating the average within each speaker. There was a main effect of the number of syllables: *F*(1, 9) = 70.700, *p* < 0.001, partial *η*^2^ = 0.887, and a main effect of break index, *F*(3, 27) = 40.139, *p* < 0.001, partial *η*^2^ = 0.817. There was no interaction between number of syllables and break index.

Bonferroni post-hoc analyses revealed that pre-boundary syllable before break 1 (*M* = 0.197, SD = 0.005) was significantly shorter than that before other break indices (break 2, *M* = 0.253, SD = 0.11, break 3, *M* = 0.261, SD = 0.008, break 4, *M* = 0.251, SD = 0.007). However, the other break indices do not differ from each other on pre-boundary syllable duration.

A two-way repeated measures ANOVA was conducted with the number of syllables in pre-boundary words (1 or more) and break index as fixed factors, and temporal distance as the dependent variable. The analysis was performed after calculating the average within each speaker. There was a main effect of number of syllables, *F*(1, 9) = 43.661, *p* < 0.001, partial *η*^2^ = 0.829, and a main effect of break index, *F*(1.157, 10.409) = 86.737, *p* < 0.001, partial *η*^2^ = 0.906.

Bonferroni post-hoc analyses showed significant difference in each pairwise comparison between temporal distance at break 1 (*M* = 0.201, SD = 0.005), break 2 (*M* = 0.309, SD = 0.016), break 3 (*M* = 0.686, SD = 0.035) and break 4 (*M* = 0.912, SD = 0.070), *p* < 0.01.

There is an interaction between number of syllables and break index, *F*(1.486, 13.370) = 10.393, *p* < 0.005, partial *η*^2^ = 0.536. A follow-up Paired-Samples *t*-Test showed that all paired samples are significantly different, *p* < 0.05. The effect of break index was more pronounced in syllables from monosyllabic words than polysyllabic words as break index increased.

#### 3.3.2. English results

In English, stress is an important factor for syllable duration. Since polysyllabic words have stressed and unstressed syllables, we report results from monosyllabic words and polysyllabic words separately. 10,345 syllables from polysyllabic words and 15,071 syllables from monosyllabic words were included in the analysis.

##### 3.3.2.1. Monosyllabic words

**Figure 17:**
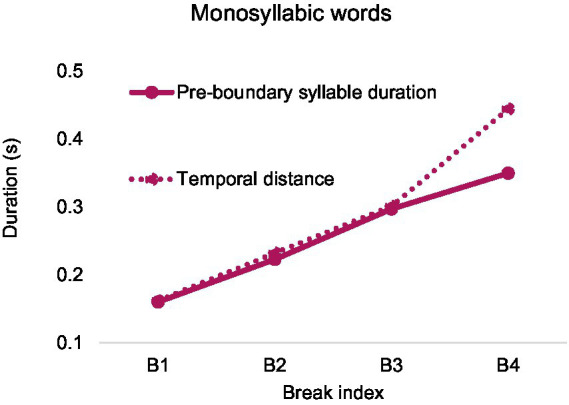
Pre-boundary syllable duration and temporal distance over break index after monosyllabic words in English.

Figure 17 shows that pre-boundary syllable duration increases gradually with break index. It also shows that temporal distance has a similar trend and is largely overlapped with pre-boundary syllable duration except for break index 4.

Repeated-measures ANOVAs on pre-boundary syllable duration and temporal distance were conducted, with break index as a fixed factor. The analysis was performed after calculating the average within each speaker. As is shown in Table 3, there are significant effects of break index on both pre-boundary syllable duration and temporal distance. Bonferroni post-hoc analyses revealed that each pairwise comparison was significant, *p* < 0.05.

**Table 3:** Results of repeated measures ANOVAs on the effect of break index on pre-boundary syllable duration and temporal distance.

Pre-boundary syllable duration	Temporal distance
*F*(3, 15) = 72.937, *p* < 0.001.	*F*(1.108, 5.540) = 38.903, *p* < 0.01.
B1 (0.160),	B1 (0.162),
B2 (0.223),	B2 (0.232),
B3 (0.297),	B3 (0.301),
B4 (0.350)	B4 (0.444)

##### 3.3.2.2. Polysyllabic words

**Figure 18:**
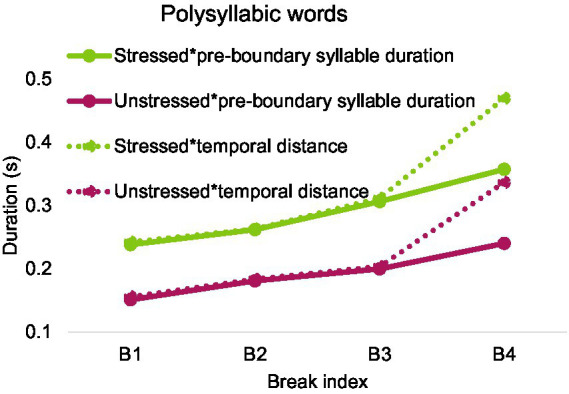
Pre-boundary syllable duration and temporal distance over break index after polysyllabic words in English.

Figure 18 shows that pre-boundary stressed and unstressed syllable duration increases gradually over break index. Also, temporal distance has a similar trend and is largely overlapped with pre-boundary syllable duration except for break index 4.

Repeated-measures ANOVAs on pre-boundary syllable duration and temporal distance were conducted with stress (stressed and unstressed) and break index as fixed factors. The analysis was performed after calculating the average within each speaker. As is shown in Table 4, there is a main effect of stress and a main effect of break index on both pre-boundary syllable duration and temporal distance. There was no interaction between the two factors. Bonferroni post-hoc analyses showed that each pairwise difference was significant, *p* < 0.05.

**Table 4:** Results of repeated measures ANOVAs on the effect of break index and stress on pre-boundary syllable duration and temporal distance in English.

	Pre-boundary syllable duration	Temporal distance
	*F*(3, 15) = 90.651,	*F*(1.117, 5.587) = 58.528,
	*p* < 0.001.	*p* < 0.001.
Break index	B1 (0.195),	B1(0.199),
	B2 (0.221),	B2(0.223),
	B3 (0.253),	B3(0.258),
	B4 (0.299)	B4(0.403)
Stress	*F*(1, 5) = 303.664,	*F*(1, 5) = 1309.778,
	*p* < 0.001	*p* < 0.001

## 4. Discussion

This study has revisited the classical rhythm class hypothesis (Pike, 1945; Abercrombie, 1967) which posits that languages of the world are either stress-timed or syllable-timed (or mora-timed). Despite failures to find evidence of equal timing at any of the alleged levels, the notion that languages are divided into timing-defined rhythm classes remains widespread and continues to drive rhythm-related research, and languages like English and Mandarin continue to be referred to as stress-timed or syllable-timed. In the last three Speech Prosody conferences alone, for example, there were 25 papers on rhythm, and 15 of them applied the rhythm metrics. A Google Scholar search on 17 December 2022 found 434 papers published in 2022 with the search term “stress-timed,” and 397 papers with the search term “syllable-timed.” In this study we have performed a more exhaustive search than before for evidence of timing-based rhythm through a corpus study aimed at identifying even the slightest tendency toward equal syllable or phrase duration in English and Mandarin, two languages that are, respectively, described as stress-timed and syllable-timed. This was done by controlling for linguistic functions that are known to significantly affect duration. We have found that there are indeed weak tendencies toward both equal syllable duration and equal phrases in Mandarin, but not in English. For English, we have found no evidence of rhythm-driven duration compensation at any level, except a weak shortening effect on consonant clusters (Figures 7–8). Once other duration-affecting factors like boundary and stress are controlled, inter-stress intervals were found to linearly vary their duration with the number of constituent syllables (Figure 9). Furthermore, phrase duration also varied linearly with their size (Figures 10, 12). This indicates that, in English, syllables are not compressible to show even a tendency toward equal inter-stress interval or equal phrase duration. The main reason for the lack of compression is the lack of flexibility of segment duration. As shown in Figures 2, 3, segments in English are also not compressible, whether measured in terms of intrinsic duration of segments (Figures 2, 3) or in terms of number of constituent segments in a syllable (Figure 6). Without such flexibility, there is no way for syllable duration to be adjustable for showing even a tendency toward equal duration of stress groups beyond timing patterns related to linguistic functions. These results have thus dissolved the central claim of the rhythm class hypothesis, namely, that English is the epitome of a stress-timed language in which the timing of the stress groups is regulated.

In contrast, Mandarin, as an alleged syllable-timed language, showed a weak tendency toward equal phrase duration (Figures 10, 13), and this tendency was grounded on the flexibility of segment duration, which also enables a tendency toward equal syllable duration. That is, syllable duration increased at a slower rate than the increase in the intrinsic segment duration (Figures 2–4) and in the number of constituent segments (Figure 6). The finding of a tendency toward equal duration of phrases runs counter to the classification of Mandarin as a syllable-timed language. This finding has demonstrated a further weakness of the rhythm class hypothesis. That is, even if it were weakened to the point of insisting on only a tendency toward isochrony, the search for such timing regularity may lead to a “wrong” language.

The results of the present study have in fact demonstrated further that, once major functional linguistic factors such as lexical stress and boundary strength (represented by break index) are taken into consideration, there is little room left for syllable, phrase or stress group duration to be further regulated by a timing control mechanism based on a purely rhythmical principle. Even the weak tendency toward equal duration of units like syllables, words and phrases in Mandarin could be explained by speakers’ inclination to devote equal amount of time resource to units of comparable levels, i.e., the tendency is motivated by functional needs rather than driven by a purely form-oriented rhythm mechanism.

Timing resource has recently been argued to be highly valuable for speech, because speech production is likely driven by a need to maximize the rate of information transmission (Xu and Prom-On, 2019). The allocation of time resource in speech is therefore likely to be balanced between various functional needs depending on their relative importance. These needs include not only those of lexical stress and boundary marking, but also the need to guarantee intelligibility of words. The intelligibility is dependent on the identifiability of their constituent segments. And the identifiability is partially determined by the functional load of segments in the language. Functional load (Hockett, 1966; Surendran and Levow, 2004) refers to the relative importance of a phonological contrast as can be calculated based on information theory (Shannon, 1948). Other things being equal, the higher the functional load of a segment, the greater the need to guarantee its intelligibility. It is also shown that the intelligibility of a segment is related to its duration, because it takes time for articulators to move to their target positions for the segment (Lindblom, 1963; Saltzman and Munhall, 1989; Perrier et al., 1996; Birkholz et al., 2011; Xu and Prom-on, 2019), and because at normal rate, speech articulation has already reached its overall maximum speed (Xu and Prom-On, 2019). Shortening syllables and hence their constituent segments beyond certain thresholds would lead to undershoot of the articulatory targets, resulting in reduced intelligibility (Cheng and Xu, 2013). To guard against excessive shortening, it would be necessary to allocate sufficient articulation time to each segment, other things being equal. So, the lack of segmental compression in a language could arise from the need to maintain segmental intelligibility.

Interestingly, it is already shown that the functional load of segments is higher in English than in Mandarin (Surendran and Levow, 2004), and this is especially true of vowels. The functional loads of consonants and vowels are 0.310 and 0.133, respectively, in English, based on Surendran and Levow’s estimation, but they are 0.235 and 0.091, respectively, in Mandarin. Most interestingly, there is a likely reason for the differences in functional load between the two languages. That is, they differ vastly in the total number of different syllables. There are only 1,268 different syllables in Mandarin with tonal differences included according to Yang and Xu (1988), or about 400 possible syllables without tonal contrast or 1,300 possible syllables with tones (Duanmu, 2000). In contrast, there are about 15,831 different syllables in English based on a count by Barker (2008). In other words, there are over 10 times as many syllables in English as Mandarin syllables with tone, or nearly 40 times as many syllables in English as Mandarin syllables without tone. To keep so many English syllables distinct from each other in speech production, it is conceivably critical that each component segment be given sufficient articulation time. In contrast, the burden of keeping only 400 Mandarin syllables distinct from each other is much lower, hence the reduced resistance to the temporal compression pressure, assuming it is present. This explanation is highly speculative, of course. But it would predict that the presence and magnitude of compression may vary across languages as a function of functional load of segments and future research could put this to test.

The present results have also demonstrated clear differences between English and Mandarin in terms of the temporal marking of boundaries of various levels. At the word level, English shows word final lengthening (Figure 12), but Mandarin does not (Figure 13). The lack of word final lengthening is surprising but interesting, as it could mean that Mandarin speech streams are not broken up at word boundaries, but only at boundaries of larger units, e.g., phrases. This possibility needs to be explored in future research. At the phrase level, there are two major differences between the two languages. At the lower phrase level, English only uses phrase-final lengthening to demarcate a phrase, whereas Mandarin also uses word medial shortening as well as shortening of the phrase final syllable relative to monosyllabic phrases (Xu and Wang, 2009) for the demarcation. For the higher phrase levels, in English pre-boundary syllable duration increases continuously with break index, whereas in Mandarin the duration increase stops beyond break index 2. This is consistent with previous reports for Mandarin (Yang, 1997; Li, 1998; Yang and Wang, 2002) and English (Wightman et al., 1992), respectively. But this is the first time that the difference between the two languages is clearly demonstrated.

One may be concerned that the duration difference between English and Mandarin is a result of the difference between the two corpora analyzed in the current study. Both corpora consist of read speech not designed for any specific experimental purposes, and they differ in that the speakers in the English corpus were professional news readers, whereas speakers in the Chinese corpus were not professional broadcasters. If any timing-based rhythm feature indeed existed, they would arguably be more easily detected from professional than from non-professional speakers. Yet the only slight equal duration tendencies were found in the speech of non-professional Mandarin speaker rather than in the speech of professional English speakers.

Nevertheless, there are a few aspects of the study that are less than ideal. One is that we did not include pitch accents as a linguistic factor in the analysis, because their annotation in the BU corpus is not independent of lexical stress (Ostendorf et al., 1995). Neither was phrasal stress considered, as it is not annotated in the corpus.

Also a potential confounding factor in the comparison of English and Mandarin temporal cues for boundary marking is the different criteria used in the labeling of the break indices between ToBI and C-ToBI. The determination of break index in English depends heavily on intonation annotation (Beckman and Ayers, 1997). Critically, break index 3 is obligatory whenever a phrase accent is present, which by definition marks the end of an intermediate phrase even if there is no silent pause. The virtual overlap of temporal distance with break index 3 in Figures 17, 18 shows that, indeed, little silence accompanied this break index. However, despite the lack of silence at break index 3 in the English corpus, significant pre-boundary lengthening was found. This indicates that English syllables are much more flexible than Mandarin in terms of lengthening beyond break index 2. On the other hand, despite the robust difference, cross-boundary temporal distance, consisting of durations of both pre-boundary syllable and silent pause, seems to be a common marker of boundary strength in both languages.

## 5. Conclusion

We have conducted a corpus study of English and Mandarin to investigate whether timing and duration in the two languages are controlled by linguistic functions or by a rhythm mechanism. The results of detailed duration analysis show that lexical stress in English and phrasing in both languages require clearly patterned durational cues for their marking, but important differences exist between the two languages. Once these functional factors are controlled for, in English, there is no further duration variation left to show any tendency toward constant duration of stress groups or phrases. In Mandarin, there is a modest tendency toward both equal syllable duration and phrase duration. These findings have largely dissolved the basic claim of the rhythm class hypothesis because English, the architype of stress-timed language has no room for constant duration of syllables, inter-stress intervals, words or phrases, and the tendency toward equal phrase duration in Mandarin violates its classification as a syllable-timed language based on rhythm metrics. We conclude, therefore, timing and duration are mainly used as cues to convey information in speech, and there is little room left for generating a rhythm pattern, and that even if the search for a cross-linguistic rhythm typology continues, the use of terms like syllable-timed and stress-timed should be avoided.

## Data availability statement

The datasets presented in this article are not readily available because they are proprietary. Access to them can be obtained from The Linguistic Data Consortium (ldc@ldc.upenn.edu) and The Institute of Linguistics Chinese Academy of Social Sciences at: http://paslab.phonetics.org.cn/?p=1763, respectively.

## Author contributions

CW and YX developed the theory. CW performed the analysis. YX supervised the project. YX provided BRC corpus and JZ provided ASCCD corpus. CW and YX wrote the manuscript with input from all authors. All authors contributed to the article and approved the submitted version.

## Funding

This research was supported by China Scholarship Council and University College London to the author CW.

## Conflict of interest

The authors declare that the research was conducted in the absence of any commercial or financial relationships that could be construed as a potential conflict of interest.

## Publisher’s note

All claims expressed in this article are solely those of the authors and do not necessarily represent those of their affiliated organizations, or those of the publisher, the editors and the reviewers. Any product that may be evaluated in this article, or claim that may be made by its manufacturer, is not guaranteed or endorsed by the publisher.
